# A pooled analysis of seven large pen feedlot studies: influence of *Megasphaera elsdenii* NCIMB 41125 on late-term mortality and growth performance of feedlot steers and heifers following terminal processing

**DOI:** 10.1093/jas/skaf263

**Published:** 2025-08-03

**Authors:** Forest L Francis, Tyler J Spore, Braden Troyer, Mark E Corrigan, David E Amrine, David G Renter, Matthew May, Calvin Booker, Tony Bryant, Josh Szasz, Alyssa Word, Kendall Karr, Ben Holland, O Abe Turgeon, Miles Theurer, Trent Fox, Zachary K Smith

**Affiliations:** Department of Animal Science & Veterinary Technology, Texas A&M University – Kingsville, Kingsville, TX 78363, USA; Axiota Animal Health, Fort Collins, CO 80528, USA; Axiota Animal Health, Fort Collins, CO 80528, USA; Axiota Animal Health, Fort Collins, CO 80528, USA; Center for Outcomes Research and Epidemiology, Kansas State University, Manhattan, KS 66502, USA; Center for Outcomes Research and Epidemiology, Kansas State University, Manhattan, KS 66502, USA; Feedlot Health, Okotoks, AB T1S2A2, Canada; Feedlot Health, Okotoks, AB T1S2A2, Canada; Five Rivers Cattle Feeding, Johnstown, CO 80534, USA; Five Rivers Cattle Feeding, Johnstown, CO 80534, USA; Cactus Research, Amarillo, TX 79101, USA; Cactus Research, Amarillo, TX 79101, USA; Cactus Research, Amarillo, TX 79101, USA; Turgeon Consulting Service, LLC, Amarillo, TX 79118, USA; Veterinary Research and Consulting Services, LLC, Hays, KS 67601, USA; Veterinary Research and Consulting Services, LLC, Hays, KS 67601, USA; Department of Animal Science, South Dakota State University, Brookings, SD 57007, USA

**Keywords:** cattle, direct-fed microbial, mortality, processing

## Abstract

Large pen feedlot studies (*n* = 7) with heifers and steers (*n* = 17,571) were conducted in North America to determine the effects of orally administered *Megasphaera elsdenii* NCIMB 41125 (Lactipro; Axiota Animal Health, Fort Collins, CO, USA) on late feeding period mortality and growth performance. Study designs were randomized complete blocks, and treatments included no administration of *Megasphaera elsdenii* NCIMB 41125 (CON) and administration of a commercial dose of *Megasphaera elsdenii* NCIMB 41125 (LP). Treatments were administered 51 to 147 d on feed to coincide with terminal implant administration and/or sorting into marketing cohorts, on subsequent mortality and growth performance. Data were analyzed with linear mixed models to account for the hierarchical design structure and fitted for mortality and performance outcomes. Mortality incidence was reduced (*P* = 0.005) when dosing *Megasphaera elsdenii* NCIMB 41125 [0.011 vs. 0.007 (per 100 head days) ± 0.002 for CON versus LP cattle, respectively]. Thus, the death rate during the risk period was 1.57 times greater in cattle not administered LP. No treatment × sex interaction (*P* ≥ 0.53) was observed for any growth performance measures. A significant effect of sex (P < 0.01) was observed for dry matter intake (DMI), average daily gain (ADG), and gain:feed (G:F), with steers exhibiting increased performance across all outcomes. No difference (*P* ≥ 0.18) between treatments was noted for DMI, ADG, or G:F. Orally administering *Megasphaera elsdenii* NCIMB 41125 to steers and heifers at terminal implant processing can be a management strategy to decrease mortality following terminal processing and during the late finishing phase.

## Teaser

Orally administering *Megasphaera elsdenii* NCIMB 41125 to steers and heifers at terminal processing can be a management strategy to decrease mortality and improve feed efficiency during the late finishing phase.

## Introduction

Mortality can be costly to beef producers and have negative impacts on the profitability of feedlot cattle. Previous research has correlated animal health to pen-level growth performance and reported that for each percentage increase in death loss, gain-to-feed ratio [average daily gain (ADG) ÷ dry matter intake (DMI); G:F] was reduced by 122 g/kg, ADG was reduced by 0.04 kg, and added cost was greater by $1.00 USD per animal in commercial Kansas feedlots (Irsik et al., 2006). Within the feedlot, the primary cause of mortality is bovine respiratory disease (BRD) with 0.41% to 0.83% of cattle placed dying annually from the disease (Miles, 2009). Following BRD, the most common cause of mortality in the feedlot is digestive disorders, with a mortality rate of 0.29% (Loneragan et al., 2001). Feeding period mortality for steers and heifers in the final 60 d before harvest has been reported between 0.16% and 0.27% (Vogel et al., 2015). Digestive mortalities occur at days on feed (DOF) approximately 22% later in the feeding period than BRD for both steers and heifers and can be costly to producers because the money invested into cattle increases as the feeding period extends (Vogel et al., 2015). By decreasing late-term digestive mortalities, producers can increase their profitability by improving cattle health, thus having more saleable cattle to market to beef processors.

*Megasphaera elsdenii* is a rumen native bacterium that preferentially ferments lactate. In cattle fed high-starch diets, *Megasphaera elsdenii* has been reported to ferment upwards of 80% of lactate in the rumen because of its preferential use of lactate compared to other known lactate-utilizing bacteria (Counotte et al., 1981). *Megasphaera elsdenii* NCIMB 41125 (Lactipro; Axiota Animal Health, Fort Collins, CO, USA) is a commercial, patented strain that displays a high growth rate (up to 0.938/h), biomass output [0.39 g (L/h)], and produces fermentation end products well below 5.5 pH. Additionally, *Megasphaera elsdenii* NCIMB 41125 is unaffected by ionophores, most in-feed anthelminthics, and antibiotics (Meissner et al., 2010), which makes this strain a notable candidate for use as a direct-fed microbial for control of lactic acidosis. The use of commercialized *Megasphaera elsdenii* NCIMB 41125 during in vitro and in vivo experiments has exhibited positive effects in the control of acidosis, as evidenced by decreased accumulation of lactic acid in the rumen and increased ruminal pH levels (Horn et al., 2009; Leeuw et al., 2016; Mazon et al., 2020). Past research has reported a 42% decrease in treatment for all disorders and a 53% decrease in treatments for diarrhea and bloat in feedlot steers orally dosed with *Megasphaera elsdenii* NCIMB 41125 (Leeuw et al., 2016). During the receiving period, high-stress calves (bulls and steers) dosed with *Megasphaera elsdenii* NCIMB 41125 had greater dry matter intake (DMI), greater average daily gain (ADG), and increased feed efficiency compared to non-dosed calves (Miller et al., 2013). Additionally, a 31% decrease in BRD-related morbidity and a 13.5% decrease in therapeutic treatment cost per calf in *Megasphaera elsdenii* NCIMB 41125 dosed calves compared to controls has been observed (Miller et al., 2013). Collectively, these findings suggest that administration of *Megasphaera elsdenii* NCIMB 41125 may enhance overall cattle health in the feedlots, indicating that large-scale commercial trials should be conducted to determine the efficacy of the product.

A pooled study analysis has many advantages over a single study analysis, primarily due to a greater sample size to compare treatment outcomes, but also because a pooled analysis provides estimated means and the associated standard errors of the means (SEM). This allows for more potential generalizability, as a pooled study analysis represents data from multiple populations of cattle, study site locations, cattle bio-type, and dietary ingredients (Smith et al., 2020). Thus, the objective of this study was to pool data from seven studies to determine the effects of orally administered *Megasphaera elsdenii* NCIMB 41125 at terminal processing on feedlot steer and heifer mortality and growth performance.

## Materials and Methods

The following research was a collaborative effort between Axiota Animal Health, Kansas State University, and South Dakota State University. Institutional Animal Care and Use Committee (IACUC) approval was not obtained at Kansas State University or South Dakota State University; all research herein was conducted at commercial research facilities and followed the guidelines stated in the Guide for the Care and Use of Agricultural Animals in Agricultural Research and Teaching (FASS, 2020).

### Trial locations, treatments, and cattle management

Large pen feedlot studies (*n* = 7 studies; *n* = 17571 cattle) conducted in North America between May 2019 and January 2022 were pooled before analysis to determine if cattle administered *Megasphaera elsdenii* NCIMB 41125 had reduced late-feeding period mortality and increased production efficiency responses following terminal processing. Studies were conducted (Table 1) using Holstein steers in Arizona (*n* = 1), mixed-breed beef steers in Kansas (*n* = 1), mixed-breed beef steers in Oregon (*n* = 1), mixed-breed beef steers in Texas (*n* = 1), mixed-breed beef heifers in Colorado (*n* = 1), mixed-breed beef heifers in Oregon (*n* = 1), and mixed-breed beef heifers in Alberta, Canada (*n* = 1). Cattle within blocks in each respective study were subjected to terminal processing, which included receiving a terminal growth-promoting implant, sorting into marketing cohorts, or both simultaneously (range of 51 to 147 d on feed). Within each study, a block was a pair of pens with a similar number and weight of cattle that received one of the two treatments administered. Treatments at terminal processing included:

**Table 1.** Descriptions of trials (*n* = 7) used in the pooled analysis.

Study	Feedlot Location	Sex/breed	Blocks (replicate pens per treatment)	Total cattle enrolled on study
1	Arizona, US	Steer/Holstein	16	4,401
2	Kansas, US	Steer/Beef	12	1,518
3	Oregon, US	Steer/Beef	6	1,779
4	Texas, US	Steer/Beef	22	3,010
5	Colorado, US	Heifer/Beef	9	2,708
6	Oregon, US	Heifer/Beef	6	1,135
7	Alberta, CA	Heifer/Beef	6	3,020
		Totals^a^	77	17,571

^a^Totals are calculated at the treatment level and not simple sums or averages of columns.

1) Not administered *Megasphaera elsdenii* NCIMB 41125 during the terminal processing event (CON)
2) Orally administered 20 or 50 mL of direct-fed microbial solution containing 1.0 × 10^10^ colony forming units of *Megasphaera elsdenii* NCIMB 41125 (Lactipro) during the terminal processing event (LP)

Following terminal processing, cattle across all studies remained on feed for an average of 84 d before shipping for harvest. Ractopamine HCl (Optaflexx; Elanco Animal Health, Indianapolis, IN, USA) was fed at an average dose of 268 mg × animal^-1^ × d^-1^ for an average of 29 d before harvest at all study sites except in Canada and Colorado. All diets fed in these experiments were based upon ingredients common to the region and contained greater than 1.38 Mcal/kg of dietary net energy for gain (NEg) and were formulated to meet or exceed requirements for protein, vitamins, and minerals for growing and finishing beef cattle (NASEM, 2016). Mortality data were obtained from each feedlot; however, the cause of mortality was not recorded at all study sites, so only overall mortality was analyzed.

Growth performance measures were calculated on a live basis and shrunk 4% to account for gastrointestinal fill. All cattle were initially weighed by pen on commercial pen scales at each feedlot, and cattle final weights were recorded by pen on either a commercial pen scale or via truck weights prior to harvest. Additionally, growth performance was calculated inclusive of cattle that died during the feeding period, as it best represents the effect of mortality on growth performance. For example, ADG was calculated as: ((Pen Final Weight—Pen Initial Weight) ÷ DOF) ÷ Number of Cattle in Pen at Study Initiation.

### Statistical analyses

Generalized linear mixed models in commercially available statistical software (R Core Team, Vienna, AT) were used for statistical analyses of feedlot mortality. Pen was the experimental unit [within block (*N* = 77) within trial (*N* = 7)]. The primary outcome variable was pen-level mortality counts. An incidence rate analysis was used to account for the fact that DOF and pen size (i.e., head-days at risk), while similar within blocks, varied across blocks and trials. Hence, Poisson and negative binomial distributions, with offsets for head-days at risk, were considered and compared using over-dispersion and information criteria. The fitted model used a Poisson distribution (log link), including treatment group, DOF quartile, and their interaction as fixed effects, and random intercepts for blocks within trials. Days on feed were categorized into quartiles as not to violate the linearity assumption necessary for fitting a continuous variable. Days on feed and the interaction term were tested as covariates but dropped from the model when not significant. Exact dates when mortalities occurred were not available for all trials; therefore, when exact mortality dates were not available, the mean approximation approach was used, whereby mortalities were assumed to occur, on average, in the middle of the trial period.

Growth performance data were analyzed with linear mixed models using the GLIMMIX procedure of SAS 9.4 (SAS Institute Inc., Cary, NC, USA). Models were fitted with a Gaussian distribution, Kenward-Roger’s denominator degrees of freedom method, pen as the experimental unit, and treatment, sex, and treatment × sex as fixed effects. Additionally, random intercepts for blocks within trials were included. Model-adjusted means and standard errors were obtained; means separations included a Tukey-Kramer adjustment for multiple comparisons. Statistical significance was denoted at an α ≤ 0.05 and tendencies were observed at 0.05 < α ≥ 0.10.

## Results and Discussion

**Figure 1.**
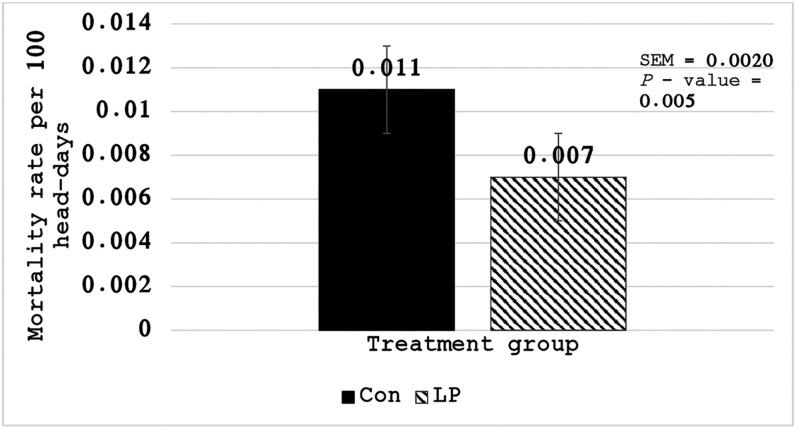
Mortality incidence rates (and SEM error bars) by treatment group (N = 7 trials). Treatments included: No *Megasphaera elsdenii* NCIMB 41125 administered during the terminal processing event (CON); *Megasphaera elsdenii* NCIMB 41125 administered during the terminal processing event (LP). Rates and SEM were results from a generalized linear mixed model using a Poisson distribution and offset of head-days, including random intercept of block within trial.

Descriptive statistics for mortality indicate that in six of the seven studies, LP cattle had decreased mortality compared to CON (Table 2). Mortality incidence rate was reduced (*P* = 0.005) in LP cattle compared to CON (0.00007% vs. 0.00011%, respectively; Figure 1). These data can be interpreted as an incidence rate ratio (0.00011/0.00007), where the death rate during the risk period was 1.57 times greater in cattle not dosed with LP. In addition, the head-days can be specified to make the results more easily interpretable (used 100 head-days in Figure 1). For example, the numbers above are equivalent to 11.0 and 7.0 (CON vs. LP respectively) mortalities per 100,000 head days; which could be interpreted as: if 2000 cattle were on feed for 50 d (100,000 head days), there would be four fewer mortalities if LP was administered at the time of the terminal processing than if no LP was administered.

**Table 2.** Descriptive statistics of trials (*n* = 7) used in the pooled analysis.

	CON^a^			LP^a^		
Study	Animals in	Study DOF	Death loss, n (%)	Animals in	Study DOF	Death loss, n (%)
1	2,201	101	12 (0.55)	2,200	101	11 (0.50)
2	761	77	5 (0.66)	757	77	7 (0.92)
3	887	65	3 (0.34)	892	65	2 (0.22)
4	1,507	51	8 (0.53)	1,503	51	3 (0.20)
5	1,355	147	56 (4.13)	1,353	147	40 (2.96)
6	567	67	6 (1.06)	568	67	2 (0.35)
7	1,509	110	28 (1.86)	1,511	109	14 (0.93)
Total^b^	8,787	84	118 (1.34)	8,784	84	79 (0.90)

^a^Treatments included: No *Megasphaera elsdenii* NCIMB 41125 administered during the terminal processing event (CON); *Megasphaera elsdenii* NCIMB 41125 administered during the terminal processing event (LP).

^b^Totals are calculated at the treatment level and not simple sums or averages of columns.

When growth performance is reported with mortalities included, researchers can evaluate how mortality rates influence cattle performance metrics, and these data can help producers make economic decisions in the feedlot. No treatment × sex interactions were observed (*P* ≥ 0.53) for DMI, ADG, or G:F, thus, only main effect means for treatment are presented. Differences were observed (*P* < 0.01) for the main effect of sex, with steers having greater DMI, ADG, and G:F (data not presented) compared to heifers. However, no differences (*P* ≥ 0.18) between CON and LP were noted for DMI, ADG, or G:F (Table 3).

**Table 3.** Production efficiency responses to LP when administered at terminal implanting^a^

Item^b^	CON^a^	LP^a^	SEM	*P*-value
DMI, lbs (kg)	10.11	10.12	0.187	0.96
ADG, lbs (kg)	1.36	1.40	0.046	0.36
G:F	0.131	0.135	0.0012	0.18

^a^Treatments included: No *Megasphaera elsdenii* NCIMB 41125 administered during the terminal processing event (CON); *Megasphaera elsdenii* NCIMB 41125 administered during the terminal processing event (LP).

^b^Growth performance was calculated inclusive of cattle that died during the feeding period, as it best represents the effect of mortality on growth performance. For example, ADG was calculated as: ((Pen Final Weight—Pen Initial Weight) ÷ DOF) ÷ Number of Cattle in Pen at Study Initiation.

Reimplantation events can be stressful on cattle, and following reimplant, it has been observed that cattle consume an average of 0.20 kg dry matter × animal^-1^ × d^-1^ less for the 10 d following processing compared to the 10 d preceding processing (Wallace et al., 2008). Periods of reduced DMI and return to greater intake can induce acidotic conditions, even in cattle consuming diets with ≥ 50% inclusion rate of forage (Zhang et al., 2013; Pederzolli et al., 2018). During periods of reduced DMI, there is a marked decrease in ruminal short-chain fatty acid (SCFA) production and a subsequent decrease in rumen papillae length, width, perimeter, and surface area (Pederzolli et al., 2018). Upon return to greater intake, SCFA production is increased, and due to reduced buffering capacity of the rumen epithelium, acid buildup occurs, and ruminal pH drops below the acidosis threshold (≈ 5.5 pH) (Zhang et al., 2013; Pederzolli et al., 2018). Additionally, periods of reduced DMI have been reported to reduce weights of regions of the gastrointestinal tract (GIT) and the liver, which implicate reduced GIT absorptive capacity and liver metabolic capacity (Lambert et al., 2023). Thus, following a reimplant event in fed steers and heifers, reduced feed intake and ruminal acidosis could lead to potential immune dysfunction. While feed intake data prior to terminal processing is unavailable for the current study pool, all cattle within the study location were subjected to the same terminal processing event and if a reduced DMI period was to occur, it could be assumed that it would affect both treatments similarly. While not statistically significant, for cattle dosed with LP, a 3.1% numerical increase in G:F was observed compared to CON. This arose from a 2.7% numerical difference in ADG for LP steers compared to CON. Previous research has reported that following a terminal implant processing event where feedlot steers were held from their home pens for 4 h and walked 0.8 km, dosing LP increased DMI for the 2 d following the event (Helmuth et al. 2022).

It has been previously reported that digestive mortalities occur at DOF 22% later in the feeding period than respiratory mortalities (Vogel et al., 2015). As DOF in the feedlot increases, it has been observed that mean ruminal pH decreases and the percentage of cattle that experience a ruminal pH of < 5.5 for more than 3 h/d increases (Castillo-Lopez et al., 2014; Cusack et al., 2021). These data indicate that as cattle are on feed for increasingly longer durations, their risk of ruminal acidosis increases, and the risk of digestive morbidity and mortality also increases. Past literature indicates that the most critical periods for the occurrence of ruminal acidosis are during the transition to high-grain diets following arrival at the feedlot and at greater DOF approaching the end of the finishing period (Nagaraja and Lechtenberg, 2007). While dosing with LP has proven beneficial for the mitigation of ruminal acidosis during transition to high-concentrate diets (Henning et al., 2010), microbial interventions for ruminal acidosis at later DOF have not been extensively researched. Later in the finishing phase, feedlot cattle exhibit their highest level of intake and have a rumen wall that has been maximally compromised due to extended periods in a low-pH environment (Nagaraja and Lechtenberg, 2007). As feedlot cattle are subjected to extended DOF, factors including severe hot and cold weather, mud, snow, laminitis, other illnesses, lameness, and fluctuation in dietary dry-matter content of rations may contribute to erratic intake patterns, which may increase the occurrences of ruminal acidosis in the herd (Nagaraja and Lechtenberg, 2007). Thus, microbial intervention with LP may help decrease the risk of acidosis at extended DOF, and as shown in the current study, reduce overall mortality following dosing at terminal processing events.

## Conclusion

Based on the pooled analysis of large pen feedlot studies, orally administered LP to steers and heifers at terminal implant processing can be a management strategy to decrease feedlot mortality during the late finishing phase. However, growth performance was not statistically enhanced, and no improvements in feed conversion efficiency were observed in LP cattle compared to non-dosed cattle. Future research trials should evaluate the effect of orally dosed LP and the effects on daily feed intake following terminal processing and various causes of morbidity and mortality during the late finishing phase in the feedlot to help determine more precisely how LP is helping to improve cattle health.

## Abbreviations

ADG: average daily gain
CON: Control
DMI: dry matter intake
DOF: days on feed
G:F: gain-to-feed ratio
GIT: gastrointestinal tract
LP: Lactipro
SCFA: short-chain fatty acid
